# Diverse Immunomodulatory Effects of Individual IFNα Subtypes on Virus-Specific CD8^+^ T Cell Responses

**DOI:** 10.3389/fimmu.2019.02255

**Published:** 2019-09-24

**Authors:** Julia Dickow, Sandra Francois, Rouven-Luca Kaiserling, Anna Malyshkina, Ingo Drexler, Astrid Maria Westendorf, Karl Sebastian Lang, Mario L. Santiago, Ulf Dittmer, Kathrin Sutter

**Affiliations:** ^1^Institute for Virology, University Hospital Essen, University of Duisburg-Essen, Essen, Germany; ^2^Institute of Virology, University Hospital Duesseldorf, Heinrich Heine University Duesseldorf, Düsseldorf, Germany; ^3^Institute of Medical Microbiology, University Hospital Essen, University of Duisburg-Essen, Essen, Germany; ^4^Institute for Immunology, University Hospital Essen, University of Duisburg-Essen, Essen, Germany; ^5^Department of Medicine, University of Colorado Denver, Aurora, CO, United States

**Keywords:** IFNα subtypes, antigen-specific CD8^+^ T cell responses, DCs, IFNAR, cytotoxicity

## Abstract

Clinical administration of Interferon α (IFNα) resulted in limited therapeutic success against some viral infections. Immune modulation of CD8^+^ T cell responses during IFNα therapy is believed to play a pivotal role in promoting viral clearance. However, these clinical studies primarily focused on IFNα subtype 2. To date, the immunomodulatory roles of the remaining 10–13 IFNα subtypes remains poorly understood, thereby precluding assessments of their potential for more effective treatments. Here, we report that virus-specific CD8^+^ T cell responses were influenced to various extents by individual IFNα subtypes. IFNα4, 6, and 9 had the strongest effects on CD8^+^ T cells, including antiproliferative effects, improved cytokine production and cytotoxicity. Interestingly, augmented cytokine responses were dependent on IFNα subtype stimulation of dendritic cells (DCs), while antiproliferative effects and cytotoxicity were mediated by IFNAR signaling in either CD8^+^ T cells or DCs. Thus, precise modulation of virus-specific CD8^+^ T cell responses may be feasible for specific antiviral immunotherapies through careful selection and administration of individual IFNα subtypes.

## Introduction

The early release of type I interferons (IFNs) is an important defense mechanism during viral infections. However, viruses have evolved many mechanisms to evade the host IFN response promoting viral replication and persistence (1). When IFN was discovered in 1957 and cloned in 1979, many virologists thought that this would be the magic bullet to treat numerous virus infections. However, 40 years later, application of exogenous type I IFNs as therapeutics is mainly restricted to the treatment of chronic infections with hepatitis B virus (HBV) and hepatitis C virus (HCV) (2, 3). With the discovery of new directly acting antiviral drugs against HCV even this therapy is now IFN-free. One possible explanation for this rather narrow area of application is the complexity of effector functions of type I IFNs, which makes it very difficult to define individual antiviral effects and mechanisms. Type I IFNs induce the expression of hundreds of IFN-stimulated genes (ISGs), some of which have direct antiviral properties, but they are also capable of modulating innate and adaptive immune responses as well as exhibiting antiproliferative activity. Several studies have tried to identify the exact molecular mechanisms of antiviral IFN therapy. Although some recent studies defined ISGs that directly interfere with the replication of specific viruses (4–6), immunomodulatory properties of type I IFNs are controversially discussed (7, 8). Modeling of HCV replication kinetics showed that during the first phase of IFN therapy, viral RNA levels rapidly declined, which was assigned to the direct antiviral effects of ISGs (9, 10). However, viral clearance through innate and adaptive immune mechanisms can only be achieved at later time points during IFNα therapy. IFN therapy in patients with human immunodeficiency virus (HIV) infection gave controversial results in terms of T cell modulation (11–14). Thus, the influence of IFNα on virus-specific immune responses is not understood at all, which hinders the development of new immunotherapies.

The complex picture becomes even more puzzling, because until now mainly data exists for IFNα2, the only clinically used subtype, but type I IFNs belong to a large family of closely related cytokines, including 12 human IFNα subtypes (14 in mice). IFNα subtypes are highly conserved proteins and bind to the same receptor, the IFNα/β receptor (IFNAR). However, they are non-redundant and have diverse biological functions (15, 16). Different binding affinities to the two IFNAR subunits (17, 18) as well as their ability to activate different downstream signaling pathways (19), which can lead to the induction of distinct ISG expression patterns for each IFNα subtype (20), are thought to dictate their individual response (21, 22). This is in line with a number of *in vitro* and *in vivo* studies, which addressed the distinct antiviral effects of individual IFNα subtypes against different viruses (23–28). However, the immunomodulatory functions of IFNα subtypes and if they differentially regulate antigen-specific CD8^+^ T cell responses were only poorly defined.

CD8^+^ T cells possess important effector functions, such as the production of cytokines or cytotoxic molecules (29, 30). Proper CD8^+^ T cell priming requires two signals (antigen recognition and co-stimulation), which are provided by professional antigen presenting cells, such as dendritic cells (DCs) (31, 32). Therefore, maturation and activation of DCs, which can be induced by cytokines, is critical to induce protective immunity against viral infections. Type I IFNs were shown to be important for optimal clonal expansion, survival, and memory formation of CD8^+^ T cells. However, these studies on the effects of type I IFNs on virus-specific T cell responses do not contain any information about the role of individual IFNα subtypes, because all human studies were performed with IFNα2 and most mouse studies were performed with an universal IFNα, a genetic hybrid of 2 human IFNα subtypes or human IFNα2.

To fully utilize the therapeutic potential of IFNα subtypes against virus infections, their immunomodulatory properties have to be defined individually. Therefore, we used the well-established Friend retrovirus (FV) mouse model to investigate the immunomodulatory potential of different murine IFNα subtypes in a standardized virus-specific proliferation assay. In preliminary *in vivo* experiments we already showed that poly I:C-induced IFNα as well as treatment with exogenous IFNα1 improved FV-specific CD8^+^ T cell responses during acute FV infection (24, 33). We now defined the effects of seven selected IFNα subtypes on the functional properties of virus-specific CD8^+^ T cells in great detail. We found that specific IFNα subtypes very potently suppressed CD8^+^ T cell proliferation, but at the same time improved their effector functions. Interestingly, IFN signaling in DCs and CD8^+^ T cells were both involved in the antiproliferative capacity of IFNα subtypes. Similar findings were made for the IFN-mediated improvement of cytotoxic responses by CD8^+^ T cells, whereas cytokine responses of CD8^+^ T cells were only augmented after IFN signaling in DCs.

## Materials and Methods

### Mice and Peptides

C57BL/6 and BALB/c mice were purchased from Harlan Laboratories (Harlan Winkelmann GmbH, Borchen, Germany) and IFNAR deficient mice (IFNAR^−/−^) (34) were kindly provided by Dr. K. S. Lang. DbGagL TCR-transgenic (tg) mice (FV TCRtg and IFNAR^−/−^ FV TCRtg) expressing an α/β-TCR specific for a H-2^b^-restricted epitope of FV GagL peptide (85-93) on CD8^+^ T cells (35, 36) and CL4 TCRtg mice expressing an α/β-TCR specific for an MHC I-restricted epitope of an influenza virus hemagglutinin (HA) (H-2Kd:HA512–520) on CD8^+^ T cells (37) were used for *in vitro* proliferation assays. Peptides derived from the FV Gag protein (sequences: CCLCLTVFL) (38) and the HA peptide (sequence: YQILAIYSTVASSLVLL) (37) were used. All mice used for experiments were at least 6 weeks of age and were followed by the ARRIVE guidelines and maintained in accordance with the regulations and guidelines of the institutional animal care and use committee of the University of Duisburg-Essen, Germany.

### Expression of IFNα Subtypes and Measurement of IFNα Activity

All IFN-encoding plasmids have been described previously (39). HEK293T cells grown in DMEM supplemented with 10% FBS were transfected with each plasmid using the calcium phosphate method. At 3 days post-transfection, supernatants were collected. Protein expression was tested using an enzyme-linked immunosorbent assay (ELISA) specific for mouse IFNα (LumiKine™ Xpress mIFN-α 2.0, Invivogen, Toulouse, France). The bioluminescent signal was assessed by the GloMax™-Multi Detection System (Promega, Madison, WI, USA). The limit of detection of IFNα was 7 pg/ml. In addition, murine IFNα subtype activity was determined by a virus-free, cell-based assay using Mx/Rage 7 cells (40). Exponentially growing cells were seeded in 96 well-plates and grown at 32°C for 24 h. Medium was removed and serial dilutions of the IFNα subtypes and commercially available recombinant mouse IFNα1, IFNα4, IFNα11, and universal IFNα (PBL assay science, Piscataway, NJ, USA) were added and cells were incubated for 24 h at 37°C. Supernatants were removed and fresh medium was added for further 48 h. Finally, cells were harvested in FACS buffer and FACS analysis was performed. 7-AAD was used to exclude dead cells. The percentage of GFP positive cells was determined and the activity of each samples was compared to the standard expressed as units/ml.

### *In vitro* Proliferation Assay and *in vitro* Kill Assay

Bone marrow derived (BM) -DCs were incubated with 0.1 μg/ml viral peptide for 90 min at 37°C. Antigen-specific CD8^+^ T cells were isolated from spleens of TCRtg mice by MACS technology (Miltenyi Biotec, Bergisch Gladbach, Germany) with a purity ≥98%, and then labeled with 5 μM CellTrace™ Violet (Thermo Scientific, Waltham, MA, USA). 2.5 × 10^5^ TCRtg CD8^+^ T cells were co-cultured with 0.5 × 10^5^ of peptide-loaded BM-DCs and stimulated with 500 units of different IFNα subtypes (1,000 units/ml). Unstimulated cells were used as controls. After 72 h of co-cultivation, proliferation of CD8^+^ T cells was assessed by flow cytometry as measured by loss of the CellTrace™ Violet dye. For the *in vitro* kill assay, 24 h after co-culturing, CFSE-labeled FBL-3 tumor cells (target cells) were added in an effector-target cell ratio of 1:1. After an additional 24 h of co-incubation, cells were resuspended in buffer containing 7-AAD for dead cell exclusion, and analyzed by flow cytometry. Percentages of dead target cells were defined as specific lysis.

### Cell Surface and Intracellular Staining by Flow Cytometry

Cell surface and intracellular staining of CD8^+^ T cells was performed as previously described (41, 42) using the following antibodies (BioLegend, San Diego, CA, USA): anti-CD8 (53-6.7), anti-GzmB (GB11), anti-IFNγ (XMG1.2), anti-IL-2 (JES6-5H4) and anti-TNFα (MP6-XT22). Dead cells were excluded from analysis (positive for fixable viability dye, Thermo Scientific). For phenotypic analysis of BM-DCs, surface staining was performed with anti-CD11b (M1/70, BioLegend), anti-CD11c (N418, Miltenyi Biotec), anti-CD80 (16-10A1, BioLegend), anti-CD86 (GL-1, BioLegend) and anti-MHC class II (M5/114.15.2, Miltenyi Biotec), and intracellular staining was performed using anti-IL-6 (MP5-20F3, BioLegend). Fluorescence minus one (FMO) controls were used for all conditions. Data were acquired on a FACS Canto II flow cytometer (BD Biosciences, Heidelberg, Germany) and analyses were performed using Flow Jo (BD Biosciences) software.

### RNA Isolation

Total RNA was isolated from splenocytes utilizing Direct-zol RNA Miniprep (Zymo Research, Freiburg, Germany). Isolated RNA was dissolved in RNase-free water and stored at −80°C.

### Real-Time-PCR

Real-time-PCR (RT-PCR) analysis for the quantification of *IL-10* mRNA (forward primer: ctggacaacatactgctaaccgactc; reverse primer: atttctgggccatgcttctctgc) was performed using PowrUp™ SYBR® Green Master Mix (Thermo Scientific). The quantitative mRNA levels were determined by using StepOne Software v2.3 (Thermo Scientific) and were normalized to β*-actin* mRNA expression levels.

### RNA Flow Cytometry

PrimeFlow RNA™ assay (Thermo Scientific) was used for single cell analysis of intracellular mRNA measured by flow cytometry. Therefore, *in vitro* proliferation assay was up-scaled to 2.5 × 10^6^ FV TCRtg CD8^+^ T cells co-cultured with 0.5 × 10^6^ of peptide-loaded BM-DCs stimulated with 500 units of IFNα4 (1,000 units/ml). Twenty-four hours later, PrimeFlow RNA™ assay was used for detection of *IL-10*-mRNA according to the manufacturer's protocol. Samples were acquired on LSR II flow cytometer (BD Biosciences) and analyses were performed using Flow Jo (Tree Star) software.

### Cytokine Detection in Cell Culture Supernatants

Bead-based LEGENDplex immunoassay for mouse Th cytokines (BioLegend) was used for the quantification of various cytokines in the co-culture supernatants. Procedures were performed according to the manufacturer's protocol. The levels of IL-10 and IL-6 were detected by using commercial ELISA Kits (BioLegend) according to the manufacturer's instructions.

### *In vivo* Cytotoxicity Assay

For the *in vivo* cytotoxicity assays, cells were prepared in accordance to the *in vitro* proliferation assay described before. Briefly, 2 × 10^6^ FV TCRtg CD8^+^ T cells (wild-type (WT) or IFNAR^−/−^) with 0.4 × 10^6^ of peptide-loaded BM-DCs (WT or IFNAR^−/−^) were adoptively transferred into IFNAR^−/−^ mice intravenously (i.v.). Mice were treated daily from day 0 to day 2 intraperitoneally (i.p.) with 8,000 units of IFNα4. At 3 days post-transfer, target cells were prepared and injected into recipient mice as previously described (41). IFNAR^−/−^ mice, which did not receive FV TCRtg CD8^+^ T cells and peptide-loaded BM-DCs, were used as naïve controls to calculate the elimination of target cells. Five hours post-transfer, recipient mice were sacrificed and cells were stained with fixable viability dye. The percentage of target-specific killing was calculated as follows: 100 – ([(% peptide pulsed CellTrace™ Violet^hi^ cells in adoptively transferred mice/% unpulsed CellTrace™ Violet^lo^ cells in adoptively transferred mice)/(% peptide pulsed CellTrace™ Violet^hi^ cells in naïve/% unpulsed CellTrace™ Violet^lo^ cells in naïve)] × 100).

### Western Blot Analysis

CD8^+^ T cells or BM-DCs were stimulated separately with 500 units of different IFNα subtypes for 15 min. Cells lysates were analyzed by Western Blots as previously described (43). Membranes were probed with specific primary antibodies (anti-p-STAT-1 [Tyr701], anti-p-STAT-2 [Tyr690], and anti-β-Actin (CST, Danvers, MA, USA)) followed by secondary antibodies coupled to peroxidase. Blots were revealed with the ECL Reagent.

### Statistical Analysis

Experimental data were reported as means +SEM. Statistically significant differences between the IFNα-treated groups and the untreated group were analyzed using Kruskal-Wallis one-way or Ordinary One Way ANOVA analysis with Dunn's multiple comparison *post-hoc* test. Statistically significant correlations were analyzed using the Pearson correlation test. Statistical analyses were performed using GraphPad Prism software (GraphPad, San Diego, CA, USA).

## Results

### IFNα Subtypes Suppress CD8^+^ T Cell Proliferation

Recent studies reported differential antiviral effects of individual mouse IFNα subtypes during viral infections (23, 24, 26). As type I IFNs regulate several hundred genes, it is nearly impossible to define their exact effects on a single immune cell population *in vivo*. To gain more insight into the regulation of CD8^+^ T cell responses by different IFNα subtypes, we analyzed the effects of murine IFNα subtypes on CD8^+^ T cell proliferation, intracellular cytokine production and cytotoxicity in a standardized *in vitro* assay. We produced and purified 7 different IFNα subtypes, which were shown to have antiviral activities (23, 24). The standard biological method to quantify the activity of IFNs is using an antiviral assays against vesicular stomatitis virus (VSV) or encephalomyocarditis virus (EMCV). However, we were concerned that the differential antiviral effects of the various interferon subtypes might produce aberrant results. Therefore, we determined the bioactivity of all IFNα subtypes in comparison to stated activities of commercially available IFNα subtypes (PBL Assay Science) in a virus-free, cell-based bioassay using Mx/Rage 7 cells, which express eGFP under the control of the Mx1 promotor (40). The percentage of eGFP-expressing cells correlates to the amount of IFNα added to the culture. All the units given in the text correspond to PBL units. PBL determines the activities of interferons using a cytopathic inhibition assay on mouse (L929) cells with EMCV.

**Figure 1:**
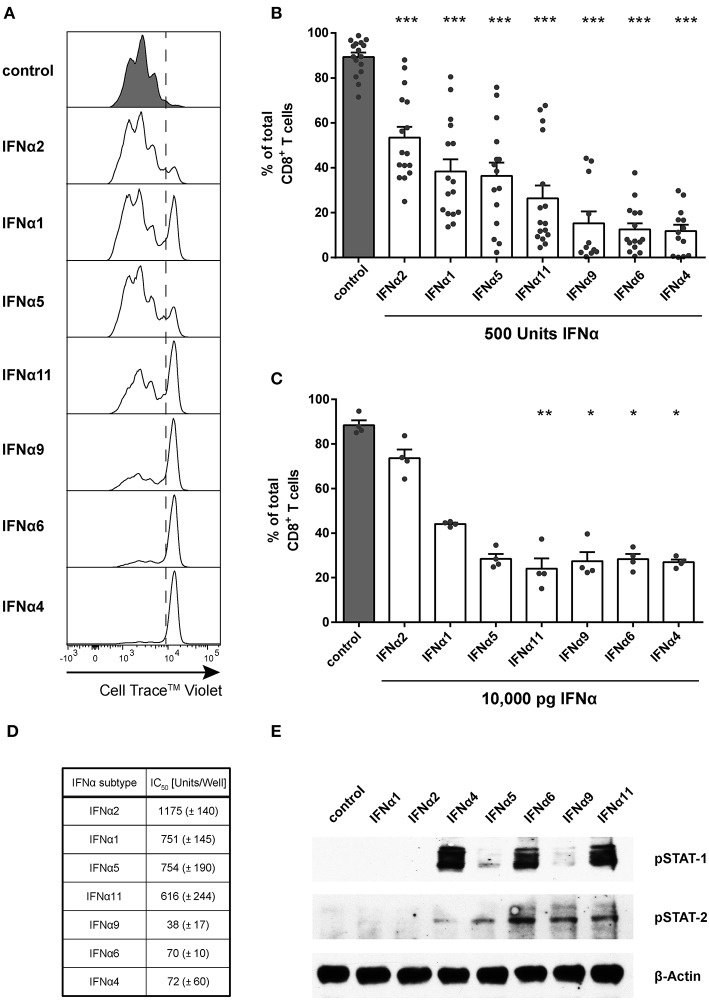
Influence of IFNα subtypes on the proliferation of FV-specific CD8^+^ T cells. Positively enriched Cell Trace^TM^ Violet-labeled CD8^+^ T cells from FV-specific TCRtg mice were co-cultured with FV peptide-loaded BM-DCs in the presence or absence of different murine IFNα subtypes for 72 h [500 units **(A,B,D,E)** or 10,000 pg **(C)**/well]. CD8^+^ T cell proliferation was measured as loss of cell tracer dye by flow cytometry. **(A)** One representative histogram is shown; the dotted line indicates the boundary between proliferating and non-proliferating cells. **(B,C)** Individual frequencies and mean values + standard error of the mean (+SEM) of proliferating CD8^+^ T cells after stimulation with 500 units of IFNα subtypes **(B)** or with 10,000 pg of IFNα subtypes **(C)** are shown as dots and bars. Statistically significant differences between IFNα subtype-stimulated cells and unstimulated cells were analyzed by Ordinary One Way ANOVA analysis and Dunn's multiple comparison and are indicated by * for *p* < 0.05; ** for *p* < 0.01; *** for *p* < 0.001. **(D)** Mean IC_50_ values (+SEM) for each IFNα subtype indicating the concentration required for 50% inhibition of proliferation are displayed in the table (*n* = 3). **(E)** Western Blot analysis of FV TCRtg CD8^+^ T cells stimulated for 15 min with 500 units of different IFNα subtypes. Antibodies against phosphorylated STAT-1 and STAT-2 and the loading control β-Actin were used as indicated.

For the *in vitro* proliferation assay, Cell Trace™ Violet-labeled FV-specific TCR transgenic (TCRtg) CD8^+^ T cells were co-cultured with BM-DCs loaded with the FV peptide that is recognized by the transgenic TCR of the CD8^+^ T cells. Without IFNα subtypes, up to 98% of all CD8^+^ T cells proliferated after 72 h of incubation (Figures 1A,B). During this time, no induction of endogenous type I IFN was detectable (neither *IFNA-*mRNA, *IFNB*-mRNA nor total IFNα protein in the supernatant; data not shown), excluding effects of endogenous type I IFNs on CD8^+^ T cell responses. Subsequently, 500 units (1,000 units/ml) of the 7 selected IFNα subtypes were added at the same time when DCs and CD8^+^ T cells were co-cultured. After stimulation with IFNα subtypes, CD8^+^ T cells proliferated significantly less (Figures 1A,B) compared to untreated cells. However, remarkable differences in the antiproliferative capacity of individual IFNα subtypes were observed and they were sorted in ascending order by their antiproliferative capacity in Figure 1B. IFNα4, IFNα6, or IFNα9 stimulation reduced the CD8^+^ T cell proliferation most potently. In contrast, treatment with IFNα2 decreased the T cell proliferation with the least efficiency. Dosages of cytokines such as IFNα subtypes are usually based on activity rather than mass units to account for the variable fraction of inactive protein that is present in recombinant preparations. However, we also repeated the experiments and stimulated the FV-specific TCRtg CD8^+^ T cells co-cultured with BM-DCs with the same protein concentration of 10,000 pg of the different murine IFNα subtypes determined by ELISA and a virus-free, cell-based assay (40) (Figure 1C). The results of both assays with either the same units or protein concentrations of IFNα subtypes gave virtually identical results in terms of their anti-proliferative activity, indicating that there is a strong correlation between biological activity and protein concentration for the individual IFNα subtypes that we produced.

To determine the IC_50_ of the antiproliferative effect of the subtypes, we used increasing concentrations of all subtypes, which reduced the CD8^+^ T cell proliferation in a concentration-dependent manner (Figure 1D). These data reveal that IFNα potently suppresses CD8^+^ T cell proliferation in a subtype- and concentration-dependent manner.

To investigate whether IFNα subtypes differentially influenced downstream signaling, we measured phosphorylation of STAT-1 and STAT-2 of IFN-stimulated CD8^+^ T cells by western blot analysis. Stimulation with all IFNα subtypes did not alter the total amount of STAT-1 and STAT-2 (data not shown). Stimulation with IFNα4, IFNα6, and IFNα11 induced a strong phosphorylation of STAT-1 in CD8^+^ T cells, while stimulation with IFNα4 and IFNα5 induced an only weak and stimulation with IFNα6, IFNα9, and IFNα11 a moderate phosphorylation of STAT-2 (Figure 1E). In contrast, stimulation with IFNα1 or IFNα2 showed no effect on the phosphorylation of STAT-1 or STAT-2 indicating that the antiproliferative potency of the different IFNα subtypes correlates with their activation of the JAK-STAT signaling pathway.

### IFNα Subtypes Improve CD8^+^ T Cell Effector Functions and Promote Target Cell Killing

**Figure 2:**
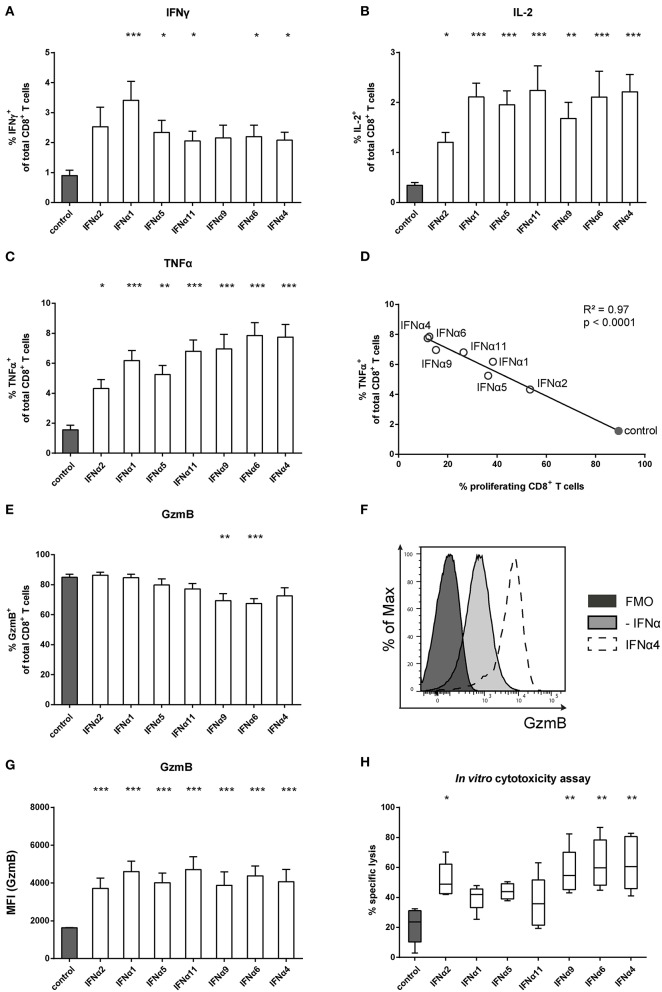
Analysis of intracellular cytokine expression of IFNα subtype-stimulated FV-specific CD8^+^ T cells. Positively enriched Cell Trace^TM^ Violet-labeled CD8^+^ T cells from FV-specific TCRtg mice were co-cultured with FV peptide-loaded BM-DCs in the presence or absence of different murine IFNα subtypes for 72 h (500 units/well). Multi-parametric flow cytometry was used to determine percentages of intracellular expression of **(A)** IFNγ, **(B)** IL-2, and **(C)** TNFα in CD8^+^ T cells (*n* = 15). Mean values (+SEM) are indicated by bars. The IFNα subtypes were sorted in the order of their antiproliferative potency. **(D)** A Pearson correlation test was used to show the correlation of the percentages of TNFα-expressing CD8^+^ T cells with the percentages of proliferating CD8^+^ T cells. Mean values of the different groups are shown as open circles (IFNα subtype-treated) or closed circle (untreated). A multi-parametric analysis of intracellular GzmB expression was performed by flow cytometry. **(E)** Percentages of GzmB^+^ CD8^+^ T cells are shown as mean values (+SEM), **(F)** a representative histogram of GzmB expression by unstimulated or IFNα4-stimulated CD8^+^ T cells and the fluorescence minus one (FMO) control is shown. **(G)** Intracellular expression of GzmB in CD8^+^ T cells analyzed by MFI (+SEM) is shown. **(H)** Cytotoxic activity of CD8^+^ T cells was analyzed in an *in vitro* cytotoxicity assay. CD8^+^ T cells and peptide-loaded BM-DCs were co-cultured for 24 h. CFSE-labeled FBL-3 target cells were added with an effector-target cell ratio of 1:1. Target cell killing was determined and dead cells were excluded by adding 7-amino actinomycin D (7-AAD). Mean values (+SEM) are indicated as box plots (*n* = 5). **(A–C,E,G,H)** Statistically significant differences between the IFNα-treated groups and the untreated group were analyzed using Kruskal-Wallis one-way or Ordinary One Way ANOVA analysis and Dunn's multiple comparison and are indicated by * for *p* < 0.05; ** for *p* < 0.01; *** for *p* < 0.001.

IFNα can also modulate immune cell functions and directly links innate and adaptive immune responses (44, 45). By providing a third signal to activated CD8^+^ T cells, IFNα ensures survival and the expression of effector molecules in T cells. We therefore investigated the role of different IFNα subtypes for the intracellular expression of cytokines, specifically IFNγ, IL-2, and TNFα, by CD8^+^ T cells. In the absence of IFNα, antigen-specific activation of CD8^+^ T cells induced low cytokine expression. Compared to untreated T cells, IFNα subtypes induced significantly higher frequencies of IFNγ, IL-2, and TNFα expressing T cells (Figures 2A–C) as well as increased expression levels measured by mean fluorescence intensity (MFI) (Figure S1). At least a two-fold increase in the percentages of IFNγ and IL-2 expressing cells was detectable after stimulation with the individual IFNα subtypes, with little variation between the groups (Figures 2A,B). In contrast, TNFα expression was more potently induced by IFNα4 and IFNα6 compared to IFNα2 (Figure 2C). The antiproliferative capacity of each IFNα subtype negatively correlated with their ability to induce TNFα in CD8^+^ T cells (Figure 2D).

To control viral infections, activated CD8^+^ T cells gain cytotoxic effector functions. We therefore tested the intracellular expression of the cytotoxic molecule GzmB in activated CD8^+^ T cells. Antigen-specific activation of CD8^+^ T cells induced massive GzmB expression in untreated CD8^+^ T cells, with more than 80% of the cells becoming GzmB-positive (Figure 2E). The frequency did not change after treatment with IFNα subtypes, except for IFNα9 and IFNα6, which slightly reduced the percentages. However, when GzmB expression levels were measured, IFNα subtype-treated CD8^+^ T cells showed higher MFI levels than untreated controls, with no obvious differences between the IFNα subtypes (Figures 2F,G). These results suggest that IFNα subtype stimulation up-regulated GzmB expression levels in activated CD8^+^ T cells. We further analyzed, whether the cytotoxic activity of CD8^+^ T cells was improved after stimulation with the different IFNα subtypes using a flow cytometry-based *in vitro* killing assay. FV-specific TCRtg CD8^+^ T cells were co-cultured with peptide-loaded BM-DCs with or without IFNα subtypes for 24 h and then FBL-3 cells, a FV-derived tumor cell line presenting FV epitopes on the surface, were added as target cells. Without IFN-stimulation, CD8^+^ T cells were able to kill about 20% of the FBL-3 cells (Figure 2H). Stimulation of CD8^+^ T cells with IFNα4 and IFNα6 resulted in a 3-fold increase in the frequencies of killed cells. In addition, IFNα2 and IFNα9 significantly enhanced CD8^+^ T cell killing, whereas stimulation with IFNα1, IFNα5 or IFNα11 had no significant effect. Taken together, these data indicate that IFNα subtype stimulation improved CD8^+^ T cell effector functions in a subtype-specific manner.

### IFNα Subtype Stimulation of CD8^+^ T Cells With Influenza Antigen Specificity

**Figure 3:**
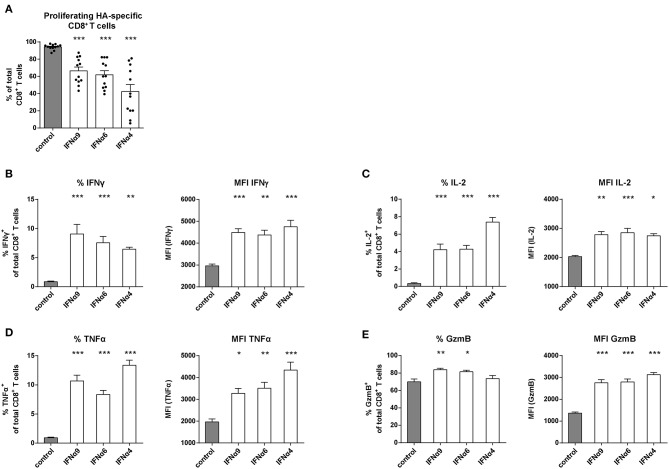
Analysis of the proliferation and intracellular cytokine production of IFNα subtype-stimulated Influenza-specific CD8^+^ T cells. Positively enriched Cell Trace^TM^ Violet-labeled CD8^+^ T cells from CL4 TCRtg mice were co-cultured with HA peptide-loaded BM-DCs in the presence or absence of IFNα4, IFNα6, or IFNα9 for 72 h (500 units/well). CD8^+^ T cell proliferation was measured as loss of cell tracer dye by flow cytometry. **(A)** Individual frequencies of proliferating CD8^+^ T cells and mean values (+SEM) are shown as dots and bars (*n* = 12). Multi-parametric flow cytometry was used to determine percentages and MFI of the intracellular expression of **(B)** IFNγ, **(C)** IL-2, **(D)** TNFα, and **(E)** GzmB in CD8^+^ T cells indicated as mean values (+SEM). Statistically significant differences between the IFNα subtype-stimulated group and the unstimulated group were analyzed by Ordinary One Way ANOVA analysis and Dunn's multiple comparison and are indicated by * for *p* < 0.05; ** for *p* < 0.01; *** for *p* < 0.001.

It was previously reported that the antiviral activity of individual IFNα subtypes depended on the type of infecting virus (46, 47). Therefore, we performed an antigen-specific CD8^+^ T cell proliferation assay using Influenza A hemagglutinin (HA)-specific TCRtg CD8^+^ T cells to mimic T cell priming during Influenza infection and ovalbumin (OVA)-specific TCRtg CD8^+^ T cells (OT-I) as one of the best characterized model antigen. We chose three IFNα subtypes (IFNα4, IFNα6 or IFNα9) that had a strong antiproliferative capacity and improved CD8^+^ T cell effector functions in the FV-specific proliferation assay. Upon antigen-specific T cell priming by peptide-loaded DCs, up to 99% of all HA-specific CD8^+^ T cells (Figure 3A) and OT-I CD8^+^ T cells (data not shown) proliferated. After treatment with IFNα4, IFNα6, or IFNα9, the proliferation of HA-specific CD8^+^ T cells was significantly reduced demonstrating the antiproliferative effect of these IFNα subtypes. IFNα4 had again the strongest antiproliferative capacity. In line with this result, in both *in vitro* assays, IFNα subtype treatment efficiently improved the effector phenotype of HA-specific CD8^+^ T cells (Figures 3B,E) and OT-I specific CD8^+^ T cells (data not shown), as depicted by the significant increase in the frequencies and expression levels (MFI) of IFNγ, IL-2, TNFα, and GzmB. These results suggest that the observed effects of IFNα subtypes on CD8^+^ T cell proliferation and effector function are not significantly influenced by the affinity of the TCR binding.

### IFNα Subtypes Induce Activation and Maturation of DCs

**Figure 4:**
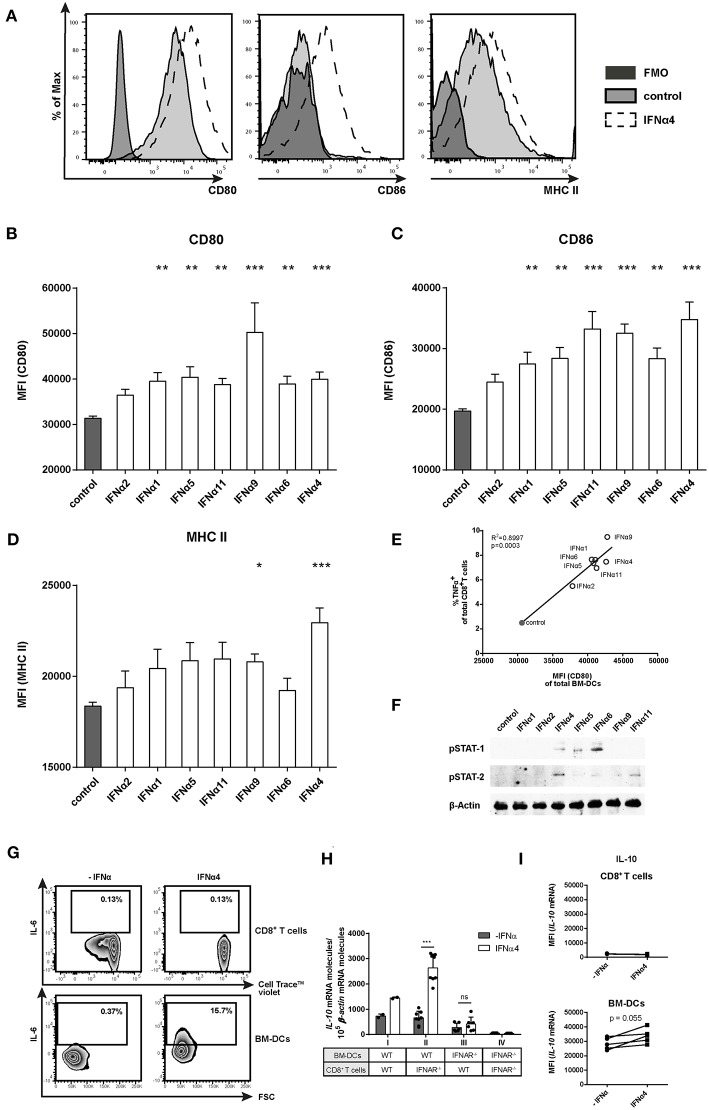
Phenotypic analysis of IFNα subtype-stimulated BM-DCs. Positively enriched Cell Trace^TM^ Violet-labeled CD8^+^ T cells from FV-specific TCRtg mice were co-cultured with FV peptide-loaded BM-DCs in the presence or absence of different murine IFNα subtypes for 72 h (500 units/well). For phenotypic characterization, BM-DCs (CD11b^+^ CD11c^+^) were analyzed by flow cytometry using anti-CD80, anti-CD86, anti-MHC class II, and anti-IL-6 antibodies. **(A)** Representative histograms of unstimulated or IFNα subtype-stimulated BM-DCs and FMOs are shown. Mean expression indicated by MFI for **(B)** CD80, **(C)** CD86, and **(D)** MHC II are shown. Mean values (+SEM) are indicated by bars. The IFNα subtypes were sorted in the order of their antiproliferative potency (*n* ≥ 9). **(E)** A Pearson correlation test was used to show the correlation of the MFI of CD80-expressing peptide-loaded BM-DCs with the percentages of TNFα-expressing CD8^+^ T cells. Mean values of the different groups are shown as open circles (IFNα subtype-treated) or closed circle (untreated) (*n* = 5). **(F)** Western Blot analysis of BM-DCs stimulated for 15 min with 500 units of different IFNα subtypes. Antibodies against phosphorylated STAT-1 and STAT-2 and the loading control β-Actin were used as indicated. **(G)** Multi-parametric flow cytometry was used to determine intracellular IL-6 expression in CD8^+^ T cells and peptide-loaded BM-DCs. Representative dot plots of untreated and IFNα4-treated co-cultures are shown. **(H)** Positively enriched CD8^+^ T cells from FV-specific TCRtg (WT) or IFNAR^−/−^ TCRtg (IFNAR^−/−^) mice were co-cultured with FV peptide-loaded WT or IFNAR^−/−^ BM-DCs in the presence or absence of IFNα4 for 24 h (500 units/well). *IL-10* mRNA expression was analyzed by RT-PCR. **(I)** Intracellular *IL-10* mRNA expression in CD8^+^ T cells and BM-DCs was determined after 24 h of co-culture by *PrimeFlow RNA*™ *Assay* and was analyzed by flow cytometry. Individual MFI of untreated and IFNα4 treated co-cultures are shown by symbols and connecting lines. **(B–D,H)** Statistically significant differences between the IFNα-treated groups and the untreated group were tested using Kruskal-Wallis one-way or Ordinary One Way ANOVA analysis and Dunn's multiple comparison and are indicated by * for *p* < 0.05; ** for *p* < 0.01; *** for *p* < 0.001.

To elucidate if the different IFNα subtypes directly modulate CD8^+^ T cell effector functions and proliferation or if these effects are mediated by the activation of BM-DCs, we analyzed the activation, maturation and cytokine profile of BM-DCs from the FV-specific proliferation assay. As shown in the representative histograms, in the absence of IFNα, BM-DCs expressed CD80 and MHC-II on their surface (Figure 4A, tinted light gray). Notably, stimulation with IFNα4 enhanced the expression of MHC-II and the co-stimulatory molecules CD80 and CD86 on BM-DCs (Figure 4A; dashed line). All tested subtypes, except IFNα2, significantly increased the expression levels of CD80 (MFI; Figure 4B) and CD86 (MFI; Figure 4C), with IFNα4, IFNα9, and IFNα11 having the strongest effect. Interestingly, the expression of MHC-II was only slightly increased after stimulation with individual IFNα subtypes and only IFNα9 and IFNα4 were able to significantly up-regulate its surface expression (Figure 4D). A significant positive correlation was detected between the percentages of TNFα-producing CD8^+^ T cells and the expression levels of CD80 on BM-DCs after stimulation with the individual IFNα subtypes (Figure 4E). Additionally, activation of the JAK-STAT signaling pathway in DCs by the different IFNα subtypes correlated with their potency to induce the expression of costimulatory molecules on BM-DCs, as stimulation with IFNα4, IFNα6, and IFNα11 induced the strongest STAT phosphorylation (Figure 4F). Next, we wanted to determine if the production of specific cytokines by BM-DCs was augmented by IFNα subtypes. For these experiments, the most potent subtype IFNα4 was used. We performed quantitative analysis for IFNγ, IL-2, TNFα, IL-6, IL-10, IL-12 (p70), IL-5, IL-13, IL-4, IL-9, IL-17a/f, IL-21, or IL-22 in supernatants from the co-cultures (data not shown). IL-2, IFNγ, and TNFα were increased after IFNα4 stimulation, correlating with the intracellular staining (Figure 2 and Figure S1). In addition, we detected IL-6 and IL-10, but none of the other cytokines mentioned above (data not shown). For these two cytokines, the concentration was strongly increased after IFNα4 treatment. Intracellular IL-6 expression in both CD8^+^ T cells and BM-DCs was analyzed by flow cytometry. Indeed, IFNα4-stimulated DCs, but not T cells, were positive for IL-6 expression (Figure 4G). To determine which cells produce IL-10 in response to IFNα4, we performed a FV-specific proliferation assay in which either CD8^+^ T cells or BM-DCs lacking the IFNAR were used (Figure 4H). *IL-10* mRNA expression was analyzed in the different groups and we observed a significant increase in *IL-10* mRNA levels in group II (IFNAR^−/−^ CD8^+^ T cells and WT BM-DCs) after stimulation with IFNα4 compared to group III (WT CD8^+^ T cells and IFNAR^−/−^ BM-DCs), in which the stimulation with IFNα4 did not change the expression of *IL-10* mRNA. From these data, we concluded that a direct IFN stimulation of DCs was responsible for the production of IL-10 in the co-cultures. To further confirm these results we analyzed the level of *IL-10* mRNA in specific cell types by a PrimeFlow RNA™ Assay (Figure 4I). Only a very low *IL-10* mRNA expression was detected in CD8^+^ T cells. In contrast, BM-DCs expressed higher levels of *IL-10* mRNA, which seemed to be further enhanced after stimulation with IFNα4. Both experiments imply a direct effect of IFNα4 on the IL-10 production by BM-DCs rather than T cells. Taken together, these results suggest that IFNα4 improved DC activation and maturation leading to an enhanced expression of the pro-inflammatory cytokine IL-6, but also the anti-inflammatory cytokine IL-10.

### DCs Play an Important Role in Mediating the Effects of IFNα Subtypes on CD8^+^ T Cells

**Figure 5:**
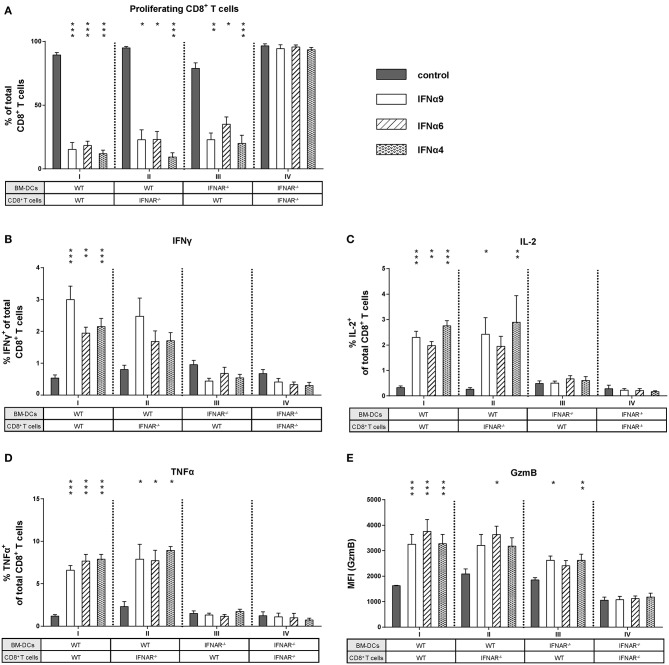
Influence of IFNAR expression on the proliferation and intracellular cytokine expression of IFNα subtype-stimulated CD8^+^ T cells. Positively enriched Cell Trace^TM^ Violet-labeled CD8^+^ T cells from FV-specific TCRtg (WT) or IFNAR^−/−^ TCRtg (IFNAR^−/−^) mice were co-cultured with FV peptide-loaded WT or IFNAR^−/−^ BM-DCs in the presence or absence of IFNα4, IFNα6, or IFNα9 for 72 h (500 units/well). **(A)** CD8^+^ T cell proliferation was measured as loss of cell tracer dye by flow cytometry and mean percentages (+SEM) of proliferating CD8^+^ T cells are shown as bars (*n* ≥ 6). Multi-parametric flow cytometry was used to determine percentages of intracellular expression of **(B)** IFNγ, **(C)** IL-2, **(D)** TNFα, and **(E)** MFI of intracellular GzmB expression in CD8^+^ T cells. Mean values (+SEM) are indicated by bars and are sorted in antiproliferative order (*n* ≥ 6). Statistically significant differences between the IFNα-treated groups and the untreated group within one approach (I–IV) were tested using Kruskal-Wallis one-way or Ordinary One Way ANOVA analysis and Dunn's multiple comparison and are indicated by * for *p* < 0.05; ** for *p* < 0.01; *** for *p* < 0.001.

As IFNα subtype stimulation improved both CD8^+^ T cell and BM-DC effector differentiation, we asked whether the observed antiproliferative and immunomodulatory effects were direct or indirect effects on CD8^+^ T cells. We performed FV-specific *in vitro* proliferation assays in which either CD8^+^ T cells or BM-DCs lacking the IFNAR were used (group I–IV). For these experiments, the most antiproliferative subtypes IFNα4, IFNα6, and IFNα9 were utilized. As expected, stimulation of WT CD8^+^ T cells and WT BM-DCs with IFNα4, IFNα6, or IFNα9 significantly reduced CD8^+^ T cell proliferation (Figure 5A, group I). In group IV, in which type I IFN signaling was absent (IFNAR^−/−^) in both cell types, the antiproliferative effect of IFNα subtypes on CD8^+^ T cells was completely abolished (Figure 5A). Interestingly, a significant antiproliferative effect of the subtypes was still observed when either IFNAR^−/−^ CD8^+^ T cells or IFNAR^−/−^ BM-DCs were used (Figure 5A, groups II and III). This indicates that IFNα can mediate suppression of T cell proliferation via direct IFNAR signaling in T cells or indirect IFNAR signaling in DCs.

Next, we wanted to elucidate whether type I IFN signaling in CD8^+^ T cells or DCs was required to improve CD8^+^ T cell effector functions after stimulation with IFNα subtypes. We analyzed cytokine expression in CD8^+^ T cells from groups I–IV. Interestingly, with DCs lacking IFNAR expression, the induction of IFNγ, IL-2, and TNFα in CD8^+^ T cells was completely abolished after IFNα subtype stimulation (Figures 5B–D, group III). In contrast, IFNAR^−/−^ CD8^+^ T cells co-cultured with WT BM-DCs still expressed IFNγ, IL-2, and TNFα in the presence of IFNα subtypes (Figures 5B–D, group II). The percentages of cytokine-positive CD8^+^ T cells were similar between group I and II and no obvious differences were found between the three cytokines analyzed. Very similar results were obtained when cytokine expression levels (MFI) in CD8^+^ T cells were determined (Figure S2). Thus, the stimulatory effect of IFNα on cytokine production by CD8^+^ T cells was mediated by an indirect mechanism involving DCs. Next, we defined the role of these two cell populations for the stimulatory effect of IFNα on CD8^+^ T cell cytotoxicity. Since IFNα subtype-stimulation had no effect on the frequency of GzmB-positive CD8^+^ T cells, we only measured GzmB expression levels (MFI). GzmB expression after IFNα stimulation was abrogated, when both CD8^+^ T cells and BM-DCs lacked the IFNAR (Figure 5E, group IV). IFNα stimulation of IFNAR^−/−^ CD8^+^ T cells and WT BM-DCs led to an increase in GzmB expression (Figure 5E, group II), which was also seen in group III, when WT CD8^+^ T cells and IFNAR^−/−^ BM-DCs were co-cultured.

Taken together, these data indicate that IFNAR expression was required solely on DCs to induce cytokine expression in CD8^+^ T cells, whereas the antiproliferative effect of IFN and an improved cytotoxic phenotype required IFNAR on both CD8^+^ T cells and DCs.

### IFNAR Signaling in CD8^+^ T Cells Is Important for Improved Target Cell Killing *in vivo*

**Figure 6:**
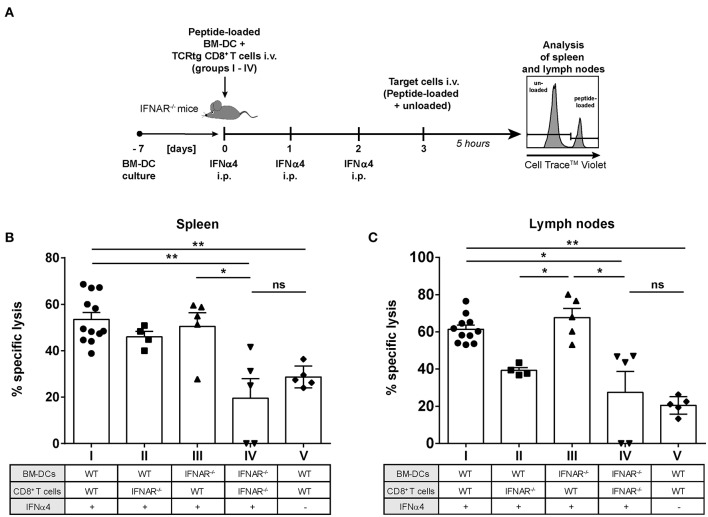
Influence of IFNAR expression on the cytotoxic activity of IFNα subtype-stimulated CD8^+^ T cells *in vivo*. Positively enriched CD8^+^ T cells from FV-specific TCRtg or IFNAR^−/−^ TCRtg mice and FV peptide-loaded WT or IFNAR^−/−^ BM-DCs (groups I–V) were adoptively transferred into IFNAR^−/−^ mice. Mice were treated daily with 8,000 units of recombinant IFNα4 starting from day 0 to day 2 post-transfer, except for group V, which did not receive any IFN treatment. At 3 days post-transfer, peptide-loaded and Cell Trace^TM^ Violet-labeled target cells (80 μM, high) were mixed with unloaded and Cell Trace^TM^ Violet-labeled target cells (2 μM, low) in a ratio of 1:1 and were injected i.v. into IFNAR^−/−^ mice. After 5 h, mice were sacrificed and the killing capacity was determined. **(A)** The experimental setup of the different groups (I–IV) and the scheme of the experimental timeline are shown. The percentages (+SEM) of target cell killing in spleen **(B)** and lymph nodes **(C)** are shown. Statistically significant differences between the groups were tested using Kruskal-Wallis one-way and Dunn's multiple comparison and are indicated by * for *p* < 0.05, ** for *p* < 0.01, and ns for not significant.

We previously demonstrated that IFNα subtype treatment increased FV-specific CD8^+^ T cell numbers *in vivo* (23, 24). Here, we showed that IFNα subtype stimulation promoted the cytotoxicity of CD8^+^ T cells *in vitro* (Figure 2H). To determine which cell population augments IFNα-driven killing by CD8^+^ T cells *in vivo*, we performed a cytotoxicity assay in the mouse, which allowed us to distinguish between the effect of IFNα subtypes on either CD8^+^ T cells or DCs. We adoptively transferred WT FV-specific TCRtg CD8^+^ T cells or IFNAR^−/−^ FV-specific TCRtg CD8^+^ T cells together with WT DCs or IFNAR^−/−^ DCs into recipient IFNAR^−/−^ mice (groups I–IV) and treated all groups with recombinant IFNα4 from day 0 to day 2 post-transfer (Figure 6A). As an additional control, we transferred WT FV-specific TCRtg CD8^+^ T cells and WT DCs into IFNAR^−/−^ mice, which were not treated with IFN (group V). At day 3, cells from naïve C57BL/6 mice were loaded with the FV GagL peptide and transferred as targets (CellTrace™ Violet^hi^) into the recipient IFNAR^−/−^ mice. Non-loaded cells (CellTrace™ Violet^lo^) were transferred as control. The elimination of target cells was analyzed after 5 h in spleen (Figure 6B) and lymph nodes (Figure 6C), as FV primarily replicates in these lymphoid organs (41).

Adoptive transfer of WT CD8^+^ T cells and WT BM-DCs (group I) induced efficient peptide-specific elimination of target cells *in vivo* after treatment with IFNα4 in both the spleen (55%) and lymph nodes (67%) (Figures 6B,C) compared to untreated controls (29% killing in spleen and 21% killing in lymph nodes; group V). Adoptive transfer of IFNAR^−/−^ CD8^+^ T cells and IFNAR^−/−^ DCs (group IV), resulted in a significant reduction of killing in spleen (20%) and lymph nodes (27%), which was similar to the untreated control (group V). IFNAR expression on CD8^+^ T cells, with BM-DCs lacking IFNAR increased the frequencies of specifically lysed target cells significantly to 50% in spleen and 67% in lymph nodes (group III). IFNAR-expressing DCs transferred with CD8^+^ T cells lacking IFNAR (group II), only partially improved elimination of targets (46% spleen and 39% lymph nodes), but the observed effect was not significant. In fact, in lymph nodes the killing in the group II mice was significantly lower than in the group III. Altogether, these data indicate that IFNAR expression on CD8^+^ T cells was critical in IFNα-mediated enhancement of the killing capacity of antigen-specific CD8^+^ T cells.

## Discussion

Exogenous application of type I IFNs in immunotherapeutic treatments represents a powerful tool against viral infections. However, the predominantly used type I IFN in the clinic is IFNα2 (48). This subtype was already used in clinical trials against HIV infection, but its therapeutic efficacy was inconclusive (12). Notably, the therapeutic potential of other IFNα subtypes remains largely untested. Here we focused on the immunomodulatory effects of different IFNα subtypes as part of the broader goal of harnessing these cytokines for therapeutic applications.

One of the earliest described functions of type I IFNs was their potential to inhibit cell division *in vitro* (49, 50), the main rationale for the use of IFNα in treating tumors (51). One study showed that the antiproliferative effect of type I IFNs depended on the phenotype of the CD8^+^ T cell. While IFNα2 promoted the expansion of human naïve CD8^+^ T cells and their differentiation into effectors, it decreased the expansion of human cytomegalovirus-specific CD8^+^ T cells (52). Notably, the same study showed no antiproliferative differences between the human subtypes IFNα2b and IFNα5. Here, we show that seven distinct mouse IFNα subtypes elicited distinct antiproliferative capacities on virus-specific CD8^+^ T cells that were activated with their cognate antigen presented by DCs (Figures 1, 3). Moreover, we found an IFNα subtype-specific antiproliferative effect on FV-specific and HA-specific CD8^+^ T cells (Figures 1, 3).

One possible explanation for the differential effects of IFNα subtypes was reported by Lavoie and colleagues, who observed that different binding affinities of the human IFNα subtypes to IFNAR correlated with their antiproliferative capacity (18). It would be interesting to determine the binding affinities of the murine type I IFNs to the murine IFNAR to correlate with antiproliferative properties. Interestingly, we show that the antiproliferative effect of IFNα on T cells was either mediated by direct IFNα stimulation of CD8^+^ T cells or indirectly mediated by DCs. This might be mediated by cell-to-cell contact or by secreted molecules, which has to be further investigated.

Many studies reported that CD8^+^ T cells contributed to the clearance of acute HBV or HCV infection (53, 54). *In vivo* depletion of CD8^+^ T cells in chimpanzees infected with HBV or HCV led to sustained high viral replication (55, 56). Accordingly, persistent HBV and HCV infection was associated with dysfunctional virus-specific CD8^+^ T cells characterized by poor proliferation, and impaired cytokine production and cytolytic activity (57, 58). Similar findings were published from studies in HIV or SIV infection (59–61). Therefore, restoration of the patient's CD8^+^ T cell response is widely considered as a promising therapy against chronic infections. Our results indicate that IFNα subtype stimulation can improve CD8^+^ T cell effector functions and their killing ability (Figures 2, 6). This is consistent with studies showing that IFNα provided a third signal to antigen-specific CD8^+^ T cells to gain cytolytic functions and the production of IFNγ (44, 52, 62). For melanoma-specific CD8^+^ T cells, it was shown that IFNα improved their cytotoxicity, while IFNα had no direct antiproliferative effect on the primary melanoma cells itself (63). Furthermore, IFNα/β, induced by acute viral infections or Poly I:C, led to sensitization of naïve (bystander) LCMV-specific CD8^+^ T cells, which were capable of upregulating the expression of cytotoxic molecules (64). However, the effects of IFNα therapy on T cells during chronic infections are still controversial (11, 12). Some studies showed that treatment with IFNα causes immune hyperactivation of CD4^+^ T cells during HIV infection, which was associated with disease progression (13). Similar findings were observed in HIV-infected humanized mice and LCMV-infected mice, in which blockage of IFNAR resulted in restoration of T cell functions and reduced viral replication (65–68).However, it was subsequently demonstrated that inhibition of IFNβ, but not IFNα, contributed to these effects in LCMV infection (69). This might explain that administration of IFNα2 in SIV-infected primates led to a significant reduction in viral loads; however, a negative effect on hyperimmune activation was not detectable (70). Another study even showed that therapy of treatment-naïve HIV-infected patients with IFNα2 contributed to increased activation of CD8^+^ T cells and reduced plasma HIV levels (14). Thus, the current studies suggest that especially IFNβ is detrimental for effective T cell responses, whereas IFNα may be used therapeutically to augment T cell responses during chronic infections. Furthermore, due to the diversity of the antiproliferative capacity of the different IFNα subtypes, distinct subtypes might not promote immune hyperactivation and thus prevent the subsequent immune dysfunction.

We previously showed that therapy with human IFNα14, but not IFNα2, led to a significant reduction of viral titers in humanized mice with an established HIV infection (27). In this study, HIV viral load reduction correlated with NK cell activation rather than CD8^+^ T cell responses. In the FV model system, treatment of acutely infected mice with the subtypes IFNα1, FNα4, IFNα9, and IFNα11 resulted in a significant reduction of viral loads, while IFNa2, IFNα5, or IFNa6 showed no anti-viral effect (23, 24). The successful treatment with different subtypes was associated with the induction of ISGs (IFNα11) (23, 24), activation of NK cells (IFNα11) (23) or improved CD8^+^ T cell responses (IFNα1) (24). Additionally, IFNα4 and IFNα5 were very potent in activating CD4^+^ and CD8^+^ T cells in the hydrodynamic injection HBV mouse model (26). Hence, careful studies may be required to determine the right IFNα subtype for optimal immunotherapy against a specific virus infection.

Proper CD8^+^ T cell activation by IFNα was in part indirectly mediated by DCs linking innate and adaptive immunity (71). DCs are professional antigen-presenting cells and migrate from sites of antigen uptake to sites of T cell activation. During this process, DCs phenotypically mature through increased expression of co-stimulatory molecules and cytokines, which are required for T cell priming and differentiation (72, 73). Interestingly, we showed that the ability of IFNα subtypes to enhance the production of pro-inflammatory cytokines in CD8^+^ T cells was fully dependent on the IFNα subtype-treated DCs (Figure 5). This is in line with another report which showed indirect effects of IFNα on CD8^+^ T cells in the LCMV model (74). In this study, the authors co-transferred WT and IFNAR^−/−^ LCMV-specific CD8^+^ T cells into mice followed by LCMV infection. The IFNAR^−/−^ LCMV-specific CD8^+^ T cells showed diminished levels of GzmB, but IFNγ and TNFα responses were unaffected. The data from IFNAR^−/−^ LCMV-specific CD8^+^ T cells further emphasize our findings that the IFNα-mediated enhancement of CD8^+^ T cell cytokine production was an exclusively indirect effect, whereas the production of cytotoxic molecules was also directly influenced by IFNAR signaling in T cells. The role of IFNα in the interaction of CD8^+^ T cells and DCs was recently investigated during infection with MVA (modified vaccinia virus Ankara)-Ova (75). In this study, the authors reported that CD8^+^ T cells expressed chemokines to attract conventional DCs and plasmacytoid DCs to the site of infection. Once arrived, pDCs produced large amounts of type I IFNs whereby conventional DCs matured, which led to optimal activation of CD8^+^ T cells. In this context, IFNAR^−/−^ CD8^+^ T cells showed no significant impairment of their effector functions, indicating the important effects of type I IFNs on the conventional DCs. However, the differential role of individual IFNα subtypes was not addressed in this study.

Taken together, our study reveals that individual IFNα subtypes have diverse impact on modulating antigen-specific CD8^+^ T cell responses. Interestingly, improved effector functions, in particular the production of IFNγ, IL-2, or TNFα, were mediated indirectly by IFNα-stimulated DCs. In contrast, antiproliferative effects and cytotoxic effector functions could be improved by IFNAR signaling in CD8^+^ T cells or DCs. These different effects of IFNα subtypes are remarkable: they improve CD8^+^ T cell effector functions but concurrently diminish their proliferative capacity resulting in lower numbers of CD8^+^ T effector cells with high potency. As CD8^+^ T cell-mediated immune protection also includes the destruction of infected cells; an uncontrolled immune response must be prevented to reduce tissue damage. IFNα subtypes likely refine antiviral T cell responses to balance immunity vs. immunopathology.

## Data Availability Statement

The datasets generated for this study are available on request to the corresponding author.

## Ethics Statement

This study was carried out in accordance with the recommendations of the Society for Laboratory Animal Science (GV-SOLAS) and the European Health Law of the Federation of Laboratory Animal Science Associations (FELASA). The protocol was approved by the North Rhine-Westphalia State Agency for Nature, Environment and Consumer Protection (LANUV), Germany (Permit Number: G1516/15).

## Author Contributions

JD and KS performed the experiments, analyzed the data, and wrote the manuscript. SF, R-LK, and AM performed experiments. ID, AW, KL, and MS contributed reagents and mice. UD interpreted the data and wrote the manuscript. All authors read and approved the final manuscript.

### Conflict of Interest

The authors declare that the research was conducted in the absence of any commercial or financial relationships that could be construed as a potential conflict of interest.
