# Supplementary material for: Diverse Immunomodulatory Effects of Individual IFNα Subtypes on Virus-Specific CD8+ T Cell Responses
Source: Front Immunol. 2019 Sep 24;10:2255. doi: 10.3389/fimmu.2019.02255 (PMC6771563; doi:10.3389/fimmu.2019.02255)
Supplement: Supplementary file 1 [file Data_Sheet_1.pdf]

Figure S1

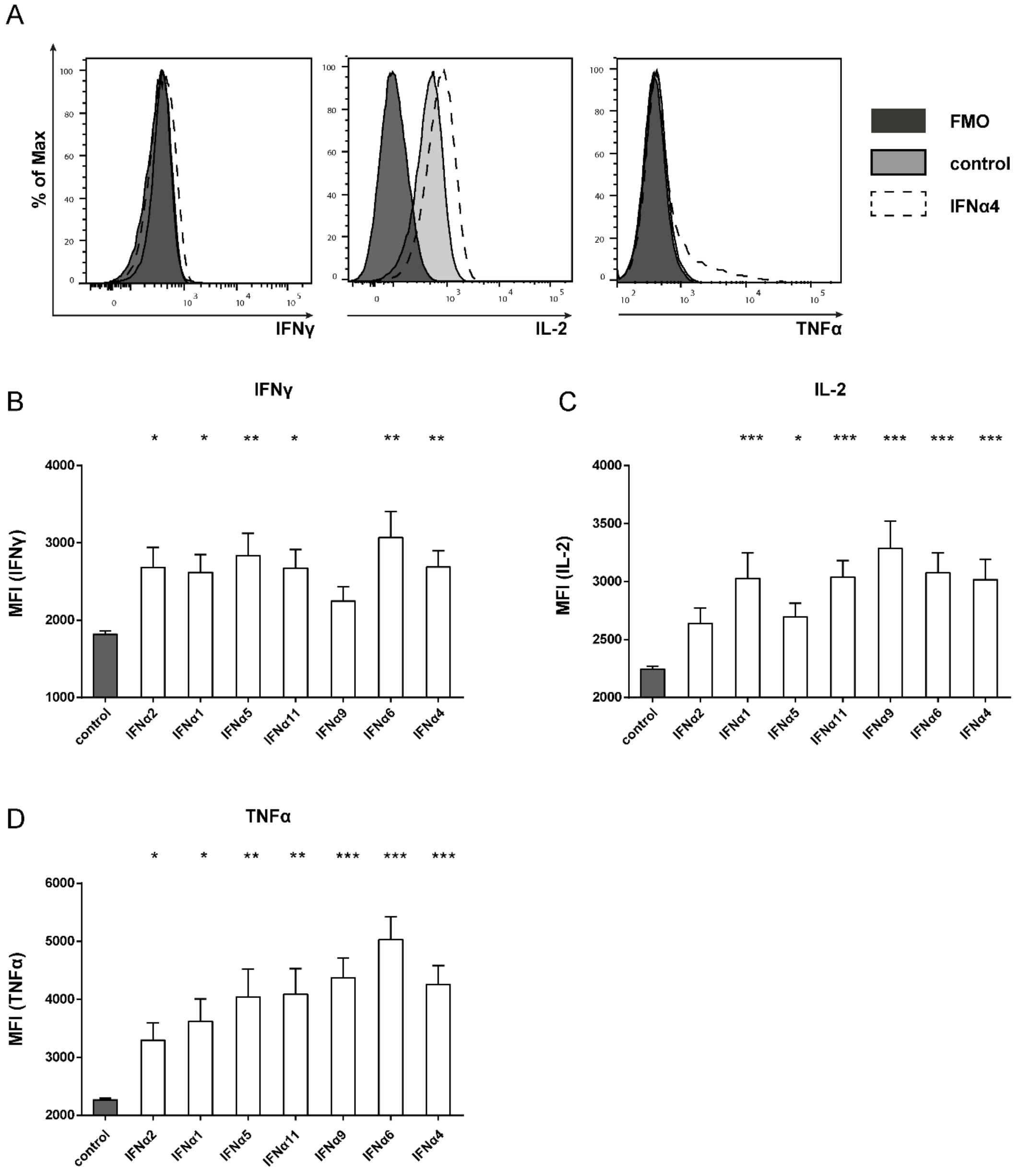

Figure S1. Analysis of intracellular cytokine expression of IFNα subtype treated CD8$^+$ T cells. Positively sorted Cell Trace^{TM} Violet-labeled CD8$^+$ T cells from FV-specific TCRtg mice were co-cultured with FV peptide-loaded BM-DCs in the presence or absence of different murine IFNα subtypes for 72 hours (500 units/well). Multi-parametric flow cytometry was used to determine MFI of intracellular expression of IFNγ, IL-2 and TNFα in CD8$^+$ T cells. (A) Representative histograms of unstimulated, IFNα4-stimulated CD8$^+$ T cells and FMOs are shown. Intracellular expression (MFI) of (B) IFNγ, (C) IL-2 and (D) TNFα are shown. Mean values (+SEM) are indicated by bars and the IFNα subtypes were sorted in the order of their antiproliferative potency (n=15). Statistically significant differences between the IFNα-treated groups and the untreated group were tested using Kruskal-Wallis one-way or Ordinary One Way ANOVA analysis and Dunn's multiple comparison and are indicated by * for $p < 0.05$; ** for $p < 0.01$; *** for $p < 0.001$.

Figure S2

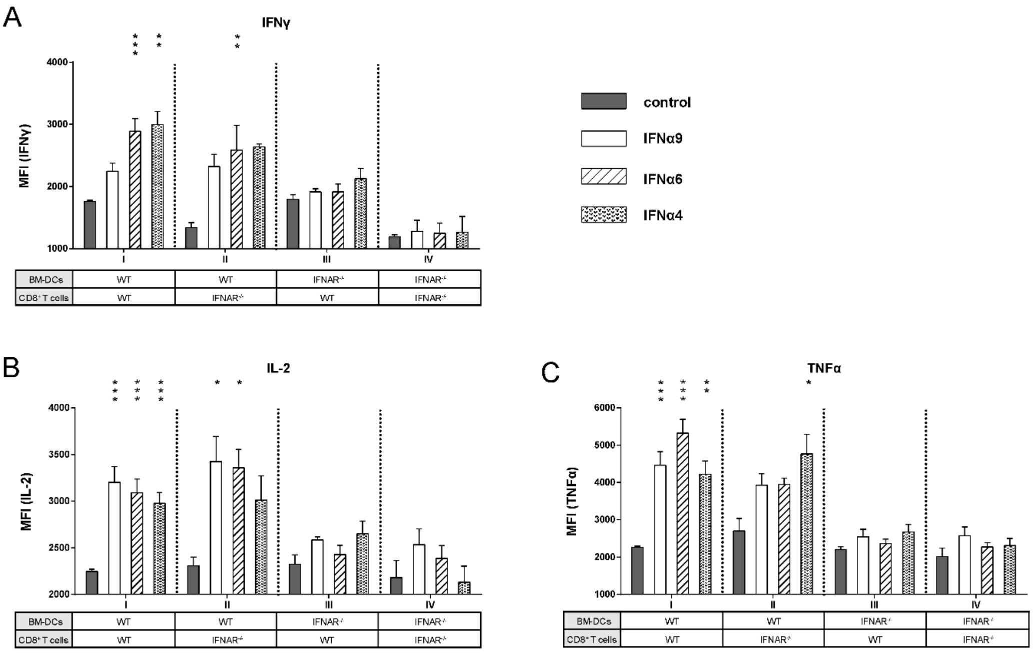

Figure S2. Influence of IFNAR expression on intracellular cytokine expression of IFNα subtype-stimulated CD8^{+} T cells. Positively enriched Cell Trace^{TM} Violet-labeled CD8^{+} T cells from FV-specific TCRtg (WT) or IFNAR$^{-/-}$ TCRtg (IFNAR$^{-/-}$) mice were co-cultured with FV peptide-loaded WT or IFNAR$^{-/-}$ BM-DCs in the presence or absence of IFNα4, IFNα6 and IFNα9 for 72 hours (500 units/well). Multi-parametric flow cytometry was used to determine MFI of intracellular expression of (A) IFNγ, (B) IL-2 and (C) TNFα in CD8^{+} T cells. Mean values (+SEM) are indicated by bars and the IFNα subtypes were sorted in the order of their antiproliferative potency (n≥6). Statistically significant differences between the IFNα-treated groups and the untreated group were tested using Kruskal-Wallis one-way or Ordinary One Way ANOVA analysis and Dunn's multiple comparison and are indicated by * for $p < 0.05$; ** for $p < 0.01$; *** for $p < 0.001$.
